# Evaluation of swallowing function in patients with oropharyngeal secretions

**DOI:** 10.1002/cre2.223

**Published:** 2019-07-23

**Authors:** Taiki Yamaguchi, Shinya Mikushi, Takao Ayuse

**Affiliations:** ^1^ Department of Clinical Physiology, Course of Medical and Dental Sciences Nagasaki University Graduate School of Biomedical Sciences Nagasaki Japan; ^2^ Department of Special Care Dentistry Nagasaki University Hospital Nagasaki Japan

**Keywords:** microaspiration, oropharyngeal secretion, saliva, swallowing function, videoendoscopy, videofluorography

## Abstract

**Background:**

Several studies have reported a strong association between the presence of oropharyngeal secretions in the laryngeal vestibule and the likelihood of aspiration of food or liquid. However, no previous studies have evaluated the accumulation of saliva and swallowing dynamics.

**Objective:**

The objective of this study was to examine the factors related to decreased function that result in saliva accumulation based on images from videofluoroscopic examination of swallowing (VF) performed on the same day as videoendoscopic examination of swallowing (VE).

**Methods:**

This retrospective study investigated 47 patients with dysphagia who underwent VF and VE on the same day. Saliva accumulation in the pharynx was assessed on VE and classified by the Murray secretion scale. Pharyngeal residue was assessed on VF. In addition, displacement of the hyoid bone and larynx on swallowing and the opening size of the esophageal orifice were measured, and contact between the base of the tongue and the posterior pharyngeal wall was examined on VF.

**Results:**

Moderate correlations were found between saliva accumulation and perpendicular displacement of the larynx and upper esophageal sphincter opening. The percentage of patients showing contact between the base of the tongue and the posterior pharyngeal wall was significantly greater in those with a saliva accumulation score of 0 or 1.

**Conclusion:**

Less laryngeal elevation and upper esophageal sphincter opening and absence of contact between the base of the tongue and the posterior pharyngeal wall when swallowing tended to result in accumulation of saliva in the pharynx.

## 1 INTRODUCTION

Although saliva in the oral cavity is an important component in swallowing, saliva also plays a critical role as a medium for carrying oral bacteria to the lower respiratory tract.(Inglis, Sherratt, Sproat, Gibson, & Hawkey, 1993) Several studies have reported a strong association between the presence of oropharyngeal secretions in the laryngeal vestibule and the likelihood of aspiration of food or liquid.(Link, Willging, Miller, Cotton, & Rudolph, 2000; Mikushi et al., 2015; Murray, Langmore, Ginsberg, & Dostie, 1996; Takahashi, Kikutani, Tamura, Groher, & Kuboki, 2012), (Takahashi et al., 2012) in a study in nursing care facilities, showed that aspiration of saliva detected by videoendoscopy was a significant risk factor for pneumonia. Silent aspiration and laryngeal penetration of saliva have been postulated to occur in the elderly(Teramoto et al., 2008) and patients with Parkinson's disease,(Ebihara et al., 2003; Gross et al., 2008; Rajaei et al., 2015) leading to aspiration pneumonia.(Kohno et al., 2013)

Decreased sensitivity of the oropharynx might be responsible for accumulation of saliva in the pharynx and subsequent silent aspiration and laryngeal penetration of saliva. However, little is known about the pathophysiological factors affecting saliva aspiration or laryngeal penetration of saliva. (Rodrigues, Nobrega, Sampaio, Argolo, & Melo, 2011) showed that the presence of hypoesthesia of the laryngeal structures and diminished protective reflexes might play major roles in the mechanisms underlying silent aspiration and silent laryngeal penetration. We have also hypothesized that reduced function of the oropharynx (i.e., incomplete pharyngeal constriction and impaired opening of the upper esophageal sphincter) may lead to accumulation or aspiration of saliva, similar to aspiration of food. No previous studies have evaluated the accumulation of saliva and swallowing dynamics. The present study examined the factors related to decreased function that result in saliva accumulation based on images from videofluoroscopic examination of swallowing (VF) performed on the same day as videoendoscopic examination of swallowing (VE).

## 2 METHODS

### 2.1 Subjects

This retrospective study investigated 47 patients with dysphagia who underwent VF and VE on the same day between October 2014 and January 2015 at Nagasaki University Hospital (Table 1). The patients were 35 men and 12 women, with a mean age of 72.8 ± 11.2 years (range, 27–97 years). Conditions underlying dysphagia were: head and neck tumor (*n* = 21), respiratory disease (*n* = 7), esophageal disease (*n* = 4), cerebrovascular disease (*n* = 3), Parkinson disease (*n* = 2), and others (*n* = 10).

**Table 1:** Patients' characteristics (n = 47)

Case No.	Sex	Age	Etiology	Lesion	Murray secretion scale(Murray et al., 1996)
1	Male	79	Head and neck tumor	Buccal mucosa	0
2	Male	97	Head and neck tumor	Buccal mucosa	0
3	Male	80	Head and neck tumor	Tongue	1
4	Male	63	Head and neck tumor	Tongue	1
5	Male	70	Head and neck tumor	Pharynx	1
6	Female	71	Head and neck tumor	Pharynx	1
7	Male	84	Head and neck tumor	Maxillary gingiva	1
8	Male	71	Head and neck tumor	Oral floor	1
9	Female	80	Head and neck tumor	Pharynx	2
10	Male	79	Head and neck tumor	Mandibular gingiva	2
11	Male	93	Head and neck tumor	Mandibular gingiva	2
12	Female	52	Head and neck tumor	Oral floor	2
13	Female	66	Head and neck tumor	Tongue	3
14	Male	66	Head and neck tumor	Tongue	3
15	Male	79	Head and neck tumor	Tongue	3
16	Male	79	Head and neck tumor	Tongue	3
17	Male	85	Head and neck tumor	Pharynx	3
18	Male	65	Head and neck tumor	Pharynx	3
19	Male	70	Head and neck tumor	Pharynx	3
20	Male	66	Head and neck tumor	Pharynx	3
21	Female	74	Head and neck tumor	Mandibular gingiva	3
22	Male	78	Respiratory disease		0
23	Male	72	Respiratory disease		0
24	Male	77	Respiratory disease		1
25	Female	73	Respiratory disease		1
26	Male	65	Respiratory disease		1
27	Male	80	Respiratory disease		1
28	Male	76	Respiratory disease		1
29	Female	77	Respiratory disease		1
30	Male	71	Respiratory disease		1
31	Male	61	Respiratory disease		3
32	Male	63	Respiratory disease		3
33	Female	69	Cerebrovascular disease	Subarachnoid hemorrhage	0
34	Male	80	Cerebrovascular disease	Putaminal hemorrhage	2
35	Male	62	Cerebrovascular disease	Medurally hemorrhage	3
36	Female	80	Parkinson disease		1
37	Male	85	Parkinson disease		1
38	Female	53	Others		0
39	Male	76	Others		0
40	Male	78	Others		0
41	Male	27	Others		0
42	Female	70	Others		0
43	Female	80	Others		0
44	Male	77	Others		1
45	Male	79	Others		3
46	Female	64	Others		3
47	Male	80	Others		3

### 2.2 VF and VE

For VF examinations, an X‐ray fluoroscopic table (C‐vision Safire L, Shimadsu, Kyoto, Japan; 30 frames/sec) was used. The examiner served test foods into each patient's mouth. Patients were asked to swallow as usual in the sitting position. Test foods were made with a barium solution (Barium: barium/water 50/50% weight/volume, Barytgen HD, Fushimi Pharmaceutical Co., Ltd., Kagawa, Japan). Thickened barium solution was made using a thickening agent (Toromelin V, Nutri, Mie, Japan). A radiopaque ball (10 mm in diameter) was attached on the left side of the neck to calibrate the image for mechanical analyses.

For VE examinations, the following systems were used (Fiberscope: FNL‐10RBS, Halogen light source: CLH‐SC, CCD camera: PSV‐4000, video system: OTV‐SC, Olympus, Tokyo, Japan). Subjects were seated comfortably in the chair. The examiner applied a topical anesthetic to the nasal mucosa and inserted the endoscope to observe saliva and the behavior of the pharynx when swallowing and coughing. Observation was performed for about 5 min. VF and VE data were recorded with a DVD recorder (DIGA, DMR‐EH50, Panasonic, Osaka, Japan) and copied onto a DVD.

### 2.3 Data collection and analysis

Patient images were collected from the VF and VE image database and analyzed using a personal computer (iMac, Apple, Cupertino, CA, USA). They were analyzed using the slow motion and stop‐frame function of Quicktime software (Version 7.76, Apple). Images were captured in JPEG format and measured by the software (Adobe Photoshop, Adobe Systems Inc., San Jose, CA, USA). Two dentists evaluated and measured the images. They had more than 8 years of clinical experience with eating and swallowing disorders, and they had training in the evaluation of dysphagia after graduation and had treated dysphagia patients. We evaluated the accumulation of saliva in the pharynx from VE, the pharyngeal residue of test food, the displacement of the hyoid bone and larynx on swallowing, the opening of the esophageal orifice, and the contact between the base of the tongue and the posterior pharyngeal wall from VF. Then, the correlations between saliva accumulation and the others were analyzed, respectively. We isolated head and neck tumor patients and analyzed them separately.

### 2.4 Accumulation of saliva in the pharynx

Saliva accumulation in the pharynx was assessed based on VE images and classified into four levels according to the method described by (Murray et al., 1996) (Table 2). To measure the reliability of the scale, interrater and intrarater reliabilities were calculated with the kappa coefficient. The interrater reliability between the two raters was 0.75, and the mean intrarater reliability was 0.83. Substantial agreement was observed in the interrater and intrarater reliabilities of this scale. Differences in their ratings were discussed by the raters to reach a consensus on the final evaluation value.

**Table 2:** The secretion severity rating scale of Murray et al.(Murray et al., 1996)

0	Most normal rating. No visible secretions anywhere in the hypopharynx or some transient bubbles visible in the valleculae and pyriform sinuses. These secretions were not bilateral or deeply pooled.
1	Any secretions evident upon entry or following a dry swallow in the channels surrounding the laryngeal vestibule that were bilaterally represented or deeply pooled. This rating would include cases where there is a transition in the accumulation of secretions during the observation segment. A subject could start with no visible secretions but accumulate secretions in an amount great enough to be bilaterally represented or deeply pooled. Likewise, a subject would be rated as a “1” if initially presenting with deeply pooled bilateral secretions and ending the observation segment with no visible secretions.
2	Any secretions that changed from a “1” rating to a “3” rating during the observation period.
3	Most severe rating. Any secretions seen in the area defined as the laryngeal vestibule. Pulmonary secretions were included if they were not cleared by swallowing or coughing at the close of the segment.

### 2.5 Pharyngeal residue of test food

Pharyngeal residue was assessed from the lateral view when 4 ml of honey‐like thickness barium were consumed for VF. The participants swallowed in accordance with the examiner's instructions. Each participant swallowed 4 ml of honey‐like thick liquid once only, and the amount of residue was evaluated after this one swallow. Swallowing dynamics were evaluated as specified by the Japanese Society of Dysphagia Rehabilitation,(Videofluoroscopic examination of swallowing, 2014) and the residue was assessed separately at the epiglottic vallecula and pyriform sinus. The evaluation scale was 1 (*large amount of residue*), 2 (*small amount of residue*), and 3 (*no residue*). Residue that occupied more than half of the capacity of the epiglottic valley and the pyriformis was defined as a large amount, whereas residue that occupied less than half of it was considered a small amount. The examiner served 4 ml of honey‐like thickness barium using a 10‐mL disposable plastic syringe. The interrater reliabilities between the two raters were 0.66 in the vallecula and 0.67 in the pyriform sinus. The mean intrarater reliabilities were 0.91 in the vallecula and 0.83 in the pyriform sinus. Substantial agreement was observed in the interrater and intrarater reliabilities of this scale. Differences in ratings were discussed by the raters to reach a consensus on the final evaluation value.

### 2.6 Displacement of the hyoid bone and larynx on swallowing

Displacement of the hyoid bone and larynx was measured on the lateral view when 4 ml of honey‐like thick liquid were swallowed for VF. The motion of the first swallowing reflex that occurred in response to the 4 ml of honey‐like thick liquid was measured. Following the method described by Logemann et al., a straight line passing through the anteroinferior borders of the second and fourth cervical vertebrae was designated as the reference line in the perpendicular direction, and displacements of the hyoid bone and larynx from the start of hyoid bone elevation to maximum elevation were measured in both horizontal and perpendicular directions.(Logemann et al., 2000) The most anterosuperior point of the body of the hyoid bone and the most anterosuperior point of the subglottic air column were selected as representative positions for the hyoid bone and larynx, respectively; they were then identified on the image, and displacement of these points was measured. Using the 10‐mm iron ball on the image as a reference, the measurement values were corrected to actual size.

### 2.7 Opening of the esophageal orifice

The opening size of the esophageal orifice was measured in the lateral view at the narrowest point of the opening between C3 and C6 during maximal distention for bolus passage when swallowing 4 ml of honey‐like thick liquid for VF.(Kendall & Leonard, 2002)

### 2.8 Contact between the base of the tongue and the posterior pharyngeal wall

**Figure 1:**
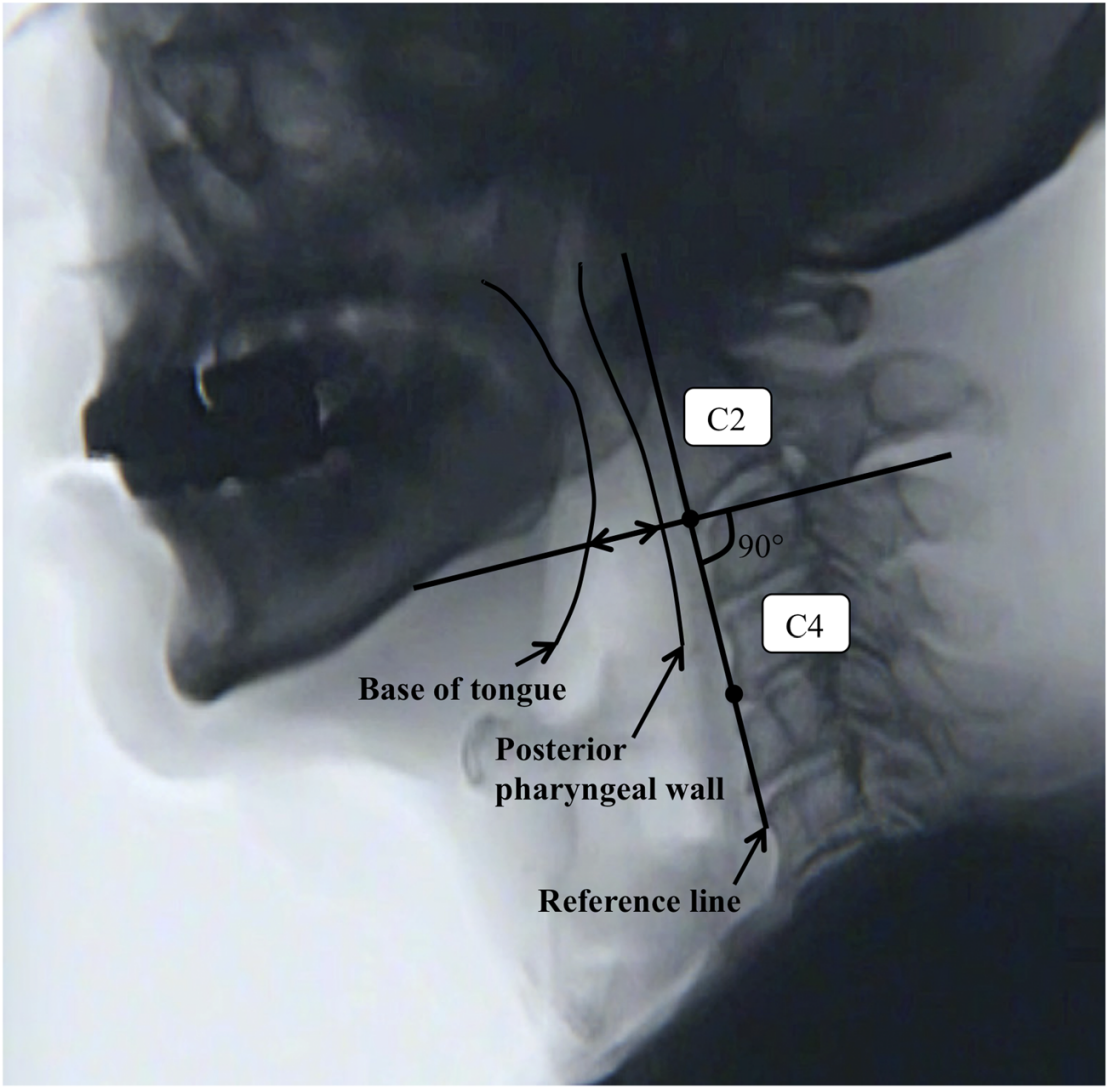
Contact between the base of the tongue and the posterior pharyngeal wall. Video image and line drawing illustrating the reference line, base of the tongue, and the posterior pharyngeal wall. Contact between the base of the tongue and the posterior pharyngeal wall was evaluated on the line that passes through the anterior inferior edge of the second cervical vertebra perpendicular to the reference line

Contact between the base of the tongue and the posterior pharyngeal wall was examined on the lateral view when 4 ml of honey‐like thick liquid were swallowed for VF. Following the method described by Fujiu and Logemann,(Fujiu & Logemann, 1996) a line connecting the anteroinferior borders of the second and fourth cervical vertebrae was designated as a reference line, and contact between the base of the tongue and the posterior pharyngeal wall at maximum elevation of the hyoid bone was assessed on the line that passes through the inferior edge of the second cervical vertebra and is perpendicular to the reference line (Figure 1). The presence or absence of contact was evaluated. Saliva accumulation was divided into those with a score of 0 or 1 and those with a score of 2 or 3, and the percentage of patients with contact was compared between these two groups.

### 2.9 Statistical methods

Statistical analyses were performed using JMP (Version 13.0, IBM, Armonk, NY, USA). Associations between saliva accumulation and each assessed value were analyzed using Spearman's rank correlation coefficients. Values of *p* < .05 were considered significant. Contact between the base of the tongue and the posterior pharyngeal wall was compared using Fisher's exact test.

### 2.10 Ethics

This study was conducted with the approval of the Ethics Committee of the Nagasaki University Hospital (approval number: 17041701). All participants gave written, informed consent before this study.

## 3 RESULTS

### 3.1 Saliva accumulation

On the basis of the evaluation method described by Murray et al,(Murray et al., 1996) the score was 0 in 11 patients (23.4%), 1 in 16 patients (34.0%), 2 in five patients (10.6%), and 3 in 15 patients (31.9%).

### 3.2 Saliva accumulation and pharyngeal residue of test food

The mean score for residue was 2.6 ± 0.6 at the epiglottic vallecula and 2.7 ± 0.6 at the pyriform sinus. Spearman's rank correlation coefficients with saliva accumulation were −.308 and −.351, respectively. There was a weak correlation between saliva accumulation and pharyngeal residue. A significant correlation with residue in the pyriform sinus was observed. In other words, pyriform sinus residue during VF increased with greater saliva accumulation and saliva penetration to the laryngeal vestibule on endoscopy.

### 3.3 Saliva accumulation and displacement of the hyoid bone and larynx on swallowing

**Figure 2:**
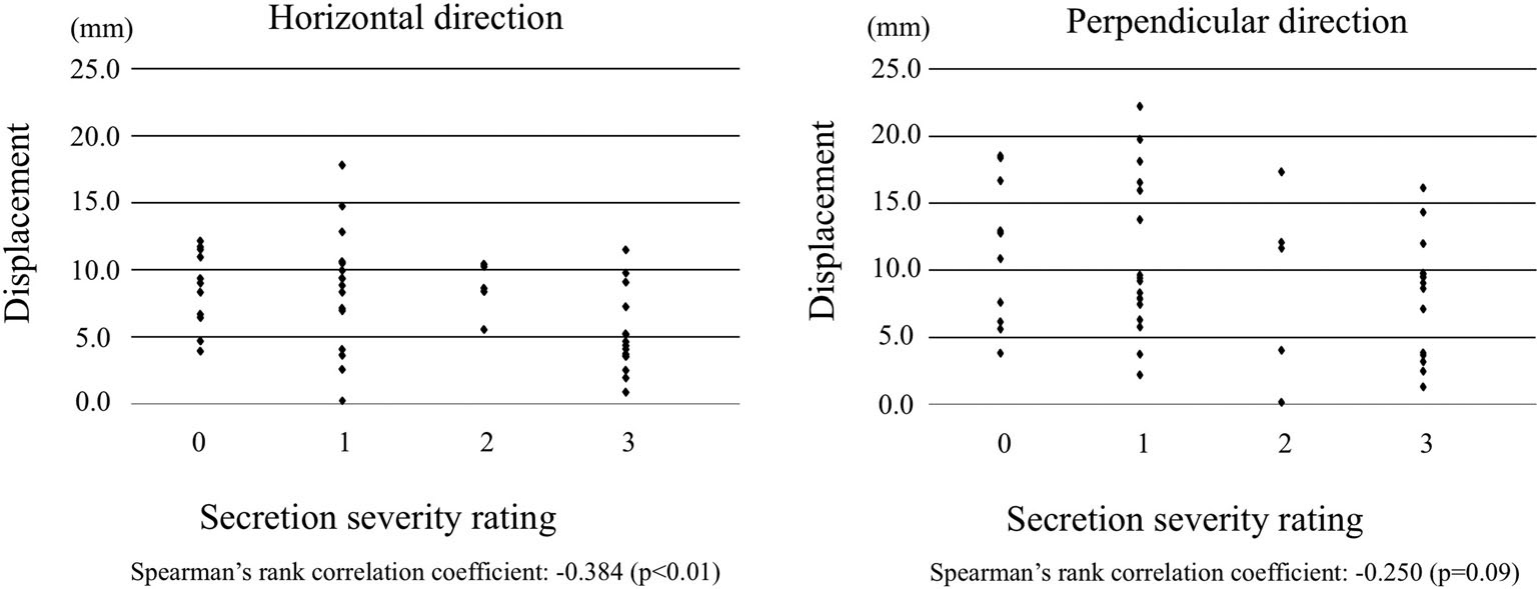
Saliva accumulation and displacement of the hyoid bone. A significant correlation between saliva accumulation and horizontal displacement of the hyoid bone is observed

**Figure 3:**
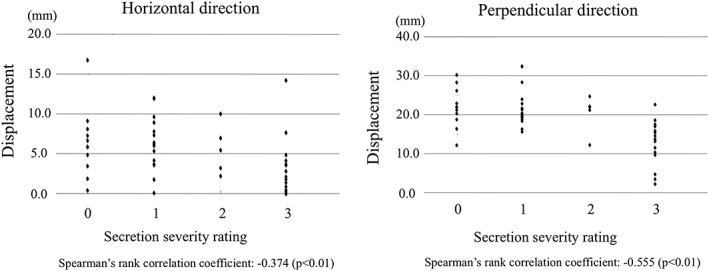
Saliva accumulation and displacement of the larynx. Significant correlations between saliva accumulation and horizontal and perpendicular displacements of the larynx are observed

Mean hyoid bone displacement on swallowing was 7.4 ± 3.8 mm in the horizontal direction and 9.9 ± 5.5 mm in the perpendicular direction. Spearman's rank correlation coefficients with saliva accumulation were −.384 and −.250, respectively. There was a weak correlation between saliva accumulation and displacement of the hyoid bone. A significant correlation with horizontal displacement of the hyoid bone was observed (Figure 2). Mean displacement of the larynx on swallowing was 5.3 ± 3.9 mm in the horizontal direction and 18.8 ± 6.7 mm in the perpendicular direction. Spearman's rank correlation coefficients with saliva accumulation were −.374 and −.555, respectively. There was a weak correlation between saliva accumulation and displacement of the larynx in the horizontal direction and a moderate correlation in the perpendicular direction. Significant correlations with both horizontal displacement and perpendicular displacement were observed (Figure 3). Patients with accumulated saliva showed small anterior displacement of the hyoid bone and small anterosuperior displacement of the larynx.

### 3.4 Saliva accumulation and opening of the esophageal orifice

**Figure 4:**
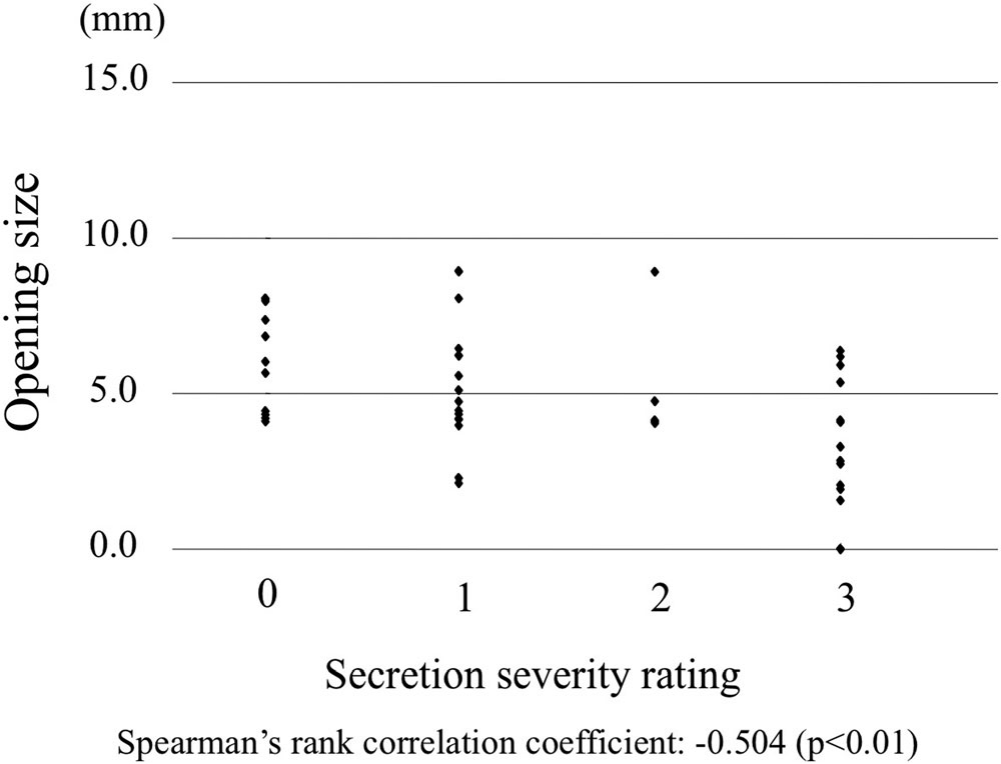
Opening of the esophageal orifice. A significant correlation between saliva accumulation and the opening size of the esophageal orifice is observed

Mean upper esophageal sphincter opening on swallowing was 5.8 ± 2.3 mm (Figure 4). Spearman's rank correlation coefficient with saliva accumulation was −.504. There was a significant moderate correlation between saliva accumulation and opening of the esophageal orifice. Patients with accumulated saliva showed smaller opening of the upper esophageal sphincter.

Saliva accumulation and contact between the base of the tongue and the posterior pharyngeal wall

Contact between the base of the tongue and the posterior wall of the pharynx was observed on swallowing in 31 patients. Specifically, contact was evident in 22 of 27 patients with a score of 0 or 1 for saliva accumulation and in nine of 20 patients with a score of 2 or 3. The percentage of patients showing contact was significantly greater in the group with a saliva accumulation score of 0 or 1 (odds ratio, 5.38; *p* = .013).

### 3.5 Head and neck tumor patients

In 21 patients with head and neck tumor, the score of Murray's secretion severity rating scale was 0 in two patients (9.5%), 1 in six patients (28.6%), 2 in four patients (19.0%), and 3 in nine patients (42.9%). The mean score for pharyngeal residue was 2.6 ± 0.6 at the epiglottic vallecula and 2.5 ± 0.7 at the pyriform sinus. Mean hyoid bone displacement on swallowing was 6.9 ± 3.2 mm in the horizontal direction and 10.1 ± 4.8 mm in the perpendicular direction. Mean displacement of the larynx on swallowing was 5.4 ± 4.0 mm in the horizontal direction and 17.8 ± 5.9 mm in the perpendicular direction. Mean upper esophageal sphincter opening on swallowing was 4.2 ± 2.5 mm. Contact between the base of the tongue and the posterior wall of the pharynx was observed on swallowing in nine patients. There was a significant moderate correlation (correlation coefficient: −.48) between saliva accumulation and displacement of the larynx in the perpendicular direction. Also, significant moderate correlation (correlation coefficient: −.57) was found between saliva accumulation and opening of the esophageal orifice.

## 4 DISCUSSION

### 4.1 Accumulation and aspiration of saliva

The combination of saliva with food that has been masticated into small pieces forms a bolus that is easily swallowed. Saliva in particular plays a key role when low‐moisture foods are consumed. In the oral cavity, countless bacteria are present and become incorporated with saliva, which is secreted not only during meals, but also at rest. Regular swallowing of saliva that has accumulated in the oral cavity and pharynx is therefore necessary. However, similar to all other fluids, there is a risk of saliva aspiration. Previously, saliva aspiration during bedtime has been reported as the phenomenon of “microaspiration.” About half of healthy individuals(Gleeson, Eggli, & Maxwell, 1997; Huxley, Viroslav, Gray, & Pierce, 1978) and 71% of patients with community‐acquired pneumonia(Kikuchi et al., 1994) show saliva aspiration while sleeping. Moreover, saliva aspiration can also occur when awake. A study that performed videoendoscopic swallowing tests in nursing home residents showed saliva aspiration in 12.1% of residents when they were awake,(Takahashi et al., 2012) and a different report showed saliva aspiration in 46% of outpatients.(Schröter‐Morasch, Bartolome, Troppmann, & Ziegler, 1999) The videoendoscopic swallowing test that we performed for inpatients also showed saliva aspiration in 34.2% of dysphagic patients.(Mikushi et al., 2015) However, although the influx of saliva into the trachea with endoscopy can be viewed in some patients while awake, endoscopic evaluation of saliva aspiration when the volume involved is too small to view is difficult. Rodrigues et al. examined saliva aspiration in patients with Parkinson's disease by applying pigment in the oral cavity.(Rodrigues et al., 2011) In particular, saliva aspiration that follows along the interarytenoid notch to the posterior wall of the trachea is difficult to evaluate due to restrictions in the angle of endoscopic viewing. For this reason, saliva accumulation, rather than aspiration, was used as the objective variable in this study. Clinically, patients with a large amount of saliva accumulation are predicted to also show saliva aspiration. (Kuo, Allen, Huang, & Lee, 2017) reported that Murray secretion scale glade correlated penetration aspiration scale strongly. Presence of pharyngeal secretion might predict patients at high risk for aspiration. Rodrigues et al. confirmed the presence of saliva aspiration in three of eight patients with saliva penetration near the glottis.(Rodrigues et al., 2011) Murray et al. report that the score was 0 in 14 patients (29%), 1 in 15 patients (32%), 2 in five patients (11%), and 3 in 13 patients (28%) in 47 hospitalized patients.(Murray et al., 1996) Their proportion was similar to this study. The score of 3 on Murray's evaluation scale in the present study signifies constant saliva accumulation in the laryngeal vestibule, and saliva aspiration may thus be occurring in these patients. In the present study, 15 of 47 patients (31.9%) had a score of 3. In the future, we plan to clarify the association between the extent of saliva accumulation and aspiration of saliva.

### 4.2 Saliva accumulation and food residue

On the basis of our experience, it appears that patients with accumulated saliva on endoscope insertion also show decreased pharyngeal function when swallowing food. The present results also showed that patients with accumulated saliva have a large amount of food residue in the pyriform sinus. Murray et al. showed that the percentage of food and liquid aspiration increases with greater severity of saliva accumulation, and that saliva accumulation is a predictor of aspiration.(Murray et al., 1996) Link et al. reported that saliva accumulation was associated with a history of pneumonia, laryngeal penetration of food, and aspiration in pediatric patients with neurologic disorders.(Link et al., 2000) In addition, we have previously reported that 46% of patients with saliva aspiration showed aspiration of honey‐like thick liquid, and that aspiration after swallowing was observed in ≥70% of aspirations observed during VE.(Mikushi et al., 2015) Eisenhuber et al. stated that 89% of patients with severe pharyngeal residue of food showed food aspiration after swallowing.(Eisenhuber et al., 2002) Food residue is also likely to be observed at the pyriform sinus in patients with accumulated saliva, indicating an elevated risk of aspiration. Patients with accumulated saliva are likely to show food aspiration after swallowing, signifying the importance of managing pyriform sinus residues and being aware of aspiration after swallowing during direct training and meals.

### 4.3 Factors that cause saliva accumulation

The accumulation of saliva has been reported to be associated with decreased laryngeal sensation and a reduced number of swallows.(Gross et al., 2008; Rajaei et al., 2015; Rodrigues et al., 2011) This is a condition in which the swallowing reflex does not become induced even when saliva accumulates in the pharynx and when swallowing should be occurring. On the other hand, saliva is predicted to remain in the pharynx also due to decreases in pharyngeal function, such as failures of pharyngeal contraction and upper esophageal sphincter opening. To the best of our knowledge, no reports have examined pharyngeal accumulation of saliva and swallowing function, but several reports have examined pharyngeal residue of food and swallowing function. Shibamoto et al. measured swallowing pressure and reported that patients who show pharyngeal residue have decreased swallowing pressure.(Shibamoto et al., 1998) Similarly, through swallowing pressure measurements in healthy elderly individuals, Dejaeger et al. found that pharyngeal residue is associated with reduced pharyngeal shortening, a low tongue force, and diminished amplitude of pharyngeal contraction.(Dejaeger, Pelemans, Ponette, & Joosten, 1997) Moreover, (Takagi, Fujishima, Ohno, et al., 2014) showed that, in ≥75‐year‐old elderly individuals, residue in the pyriform sinus was associated with decreased tongue pressure. Olsson et al. reported that patients who show pharyngeal residue of food have a small opening size of the upper esophageal sphincter and a small amount of laryngeal elevation.(Olsson, Castell, Johnston, Ekberg, & Castell, 1997)

The results of the present study showed that saliva accumulation was associated with perpendicular displacements of the larynx, contact between the base of the tongue and the posterior wall of the pharynx, and opening size of the upper esophageal sphincter. Perpendicular displacements of the larynx and opening size of the upper esophageal sphincter were also associated with saliva accumulation in head and neck tumor patients. (Logemann et al., 2000) reported that healthy older individuals showed hyoid bone displacement of 8.47 ± 1.05 mm in the horizontal direction and 14.58 ± 1.46 mm in the perpendicular direction and laryngeal displacement of 6.18 ± 0.93 mm in the horizontal direction and 24.25 ± 1.58 mm in the perpendicular direction. All of these values were larger than the present findings; we postulated that this was due to the inclusion of dysphagia patients in the present study. Regarding upper esophageal sphincter opening, (Kendall & Leonard, 2002) reported that in ≥65‐year‐old dysphagia patients, an opening of 7.6 ± 0.27 mm was observed with 1 ml of liquid and 8.1 ± 0.31 mm with 20 ml of liquid. Hattori et al.(Hattori, 2004) reported that in healthy older individuals, an opening of 8.18 ± 2.12 mm was observed with the consumption of 8 ml of paste. In the present study, 4 ml of paste were used, and the mean opening was 5.8 ± 2.3 mm, indicating a smaller opening compared with the above reports.

Upper esophageal sphincter opening requires relaxation of the sphincter and mechanical opening through pharyngeal pressure and laryngeal elevation on swallowing. The present results demonstrated that many patients with accumulated saliva did not show contact between the base of the tongue and the posterior wall of the pharynx on swallowing, indicating a deficiency in pharyngeal pressure. In the future, we plan to conduct manometry and measure tongue pressure to confirm this finding of insufficient pharyngeal pressure on swallowing. In addition, deficient laryngeal elevation are linked to deficient upper esophageal sphincter opening, causing saliva accumulation similarly to food accumulation. These findings indicated that saliva accumulation is associated not only with decreased sensation, but also with decreased pharyngeal function. To reduce saliva accumulation, improvement of laryngeal elevation, pharyngeal contraction, and upper esophageal sphincter opening is crucial, similar to the approach for reducing food residue in the pharynx.

### 4.4 Limitations of the study

The limitations of this study are that it was a retrospective evaluation, used only one thick‐liquid swallowing measurement that was performed during a clinical examination, and included a small sample size, resulting in poor reliability. With a larger sample size, comparisons by sex, age, and underlying disease can be carried out. We therefore plan to increase the number of study participants in the future. The evaluation of residue was conducted using a method that is used only within Japan. Furthermore, all study participants were Japanese; thus, there may also be differences in measurements based on ethnicity.

## 5 CONCLUSION

Pharyngeal malfunction was associated with saliva accumulation in the pharynx. The tendencies of pharyngeal function that may lead to saliva accumulation included: small vertical laryngeal displacement during swallowing, small opening of the esophageal orifice, and absence of contact between the base of the tongue and the posterior pharyngeal wall.

## CONFLICT OF INTEREST

The authors declare that they do not have any conflict of interest.
